# Functional and structural specific roles of activity-driven BDNF within circuits formed by single spiny stellate neurons of the barrel cortex

**DOI:** 10.3389/fncel.2014.00372

**Published:** 2014-11-06

**Authors:** Qian-Quan Sun, Zhi Zhang, June Sun, Anand S. Nair, Dan P. Petrus, Chunzhao Zhang

**Affiliations:** Department of Zoology and Physiology, University of Wyoming, Laramie, WYUSA

**Keywords:** variance–mean analysis, neurotrophin, dendritic spine, axonal arborizations, presynaptic

## Abstract

Brain derived neurotrophic factor (BDNF) plays key roles in several neurodevelopmental disorders and actions of pharmacological treatments. However, it is unclear how specific BDNF’s effects are on different circuit components. Current studies have largely focused on the role of BDNF in modification of synaptic development. The precise roles of BDNF in the refinement of a functional circuit *in vivo* remain unclear. Val66Met polymorphism of BDNF may be associated with increased risk for cognitive impairments and is mediated at least in part by activity-dependent trafficking and/or secretion of BDNF. Using mutant mice that lacked activity-driven BDNF expression (*bdnf*-KIV), we previously reported that experience regulation of the cortical GABAergic network is mediated by activity-driven BDNF expression. Here, we demonstrate that activity-driven BDNF’s effects on circuits formed by the layer IV spiny stellate cells are highly specific. Structurally, dendritic but not axonal morphology was altered in the mutant. Physiologically, GABAergic but not glutamatergic synapses were severely affected. The effects on GABA transmission occurs via presynaptic alteration of calcium-dependent release probability. These results suggest that neuronal activity through activity-driven BDNF expression, can selectively regulate specific features of layer IV circuits *in vivo*. We postulate that the role of activity-dependent BDNF is to modulate the computational ability of circuits that relate to the gain control (i.e., feed-forward inhibition); whereas the basic wiring of circuits relevant to the sensory pathway is spared. Gain control modulation within cortical circuits has broad impact on cognitive processing and brain state-transitions. Cognitive behavior and mode is determined by brain states, thus the studying of circuit alteration by endogenous BDNF provides insights into the cellular and molecular mechanisms of diseases mediated by BDNF.

## INTRODUCTION

Brain derived neurotrophic factor (BDNF) plays key roles in several neurodevelopmental and neuropsychiatric disorders including Rett Syndrome (Chang et al., 2006; Li and Pozzo-Miller, 2014), schizophrenia (Durany and Thome, 2004; Lu and Martinowich, 2008), major depression disorder (MDD; Martinowich et al., 2007), attention deficit hyperactivity disorder (ADHD; Kebir et al., 2009; Caylak, 2012), and actions of pharmacological treatments (Longo and Massa, 2013; Ninan, 2014) in these diseases. A consensus of the role of BDNF in neurodevelopmental disorders is related to its role in the regulation of synaptic maturation within critical brain areas (Li and Pozzo-Miller, 2014). Current studies have largely focused on the role of BDNF in modification of synaptic development (Lu, 2003; English et al., 2012). The precise roles of BDNF in the refinement of an entire functional circuit *in vivo* remain unclear.

The refinement of a neural circuit during development depends on a dynamic process of axonal and dendritic branching that leads to changes in synaptic connectivity. Neuronal activities play a crucial role in neural circuit refinement. It has been proposed that BDNF acts as a modulator, rather than a direct mediator of activity during the morphological development of neural circuits (Cohen-Cory, 1999). The transcription of the *bdnf* gene is mediated by nine discrete promoters; each driving a unique 5′ exon (exons I–VIII) that is spliced onto the common 3′ coding exon (exon IX) to synthesize the same pre-, pro- BDNF proteins (Aid et al., 2007). These promoters drive activity-, epigenetic-, or hormonal -dependent BDNF expressions. Single nucleotide polymorphism in BDNF val66met allele is implicated with increased risk for schizophrenia, and cognitive impairments and is mediated at least in part by activity-dependent trafficking and/or secretion of BDNF (Lu and Martinowich, 2008; Li and Pozzo-Miller, 2014). Although extensive knowledge has been accumulated regarding the roles of BDNF signaling at the organismal and regional tissue level, relatively little is known about BDNF expression being driven by different promoters (e.g., activity-dependent endogenous BDNF), in playing a specific roles in the formation of a functional circuit *in vivo*. There are examples where BDNF can differentially modulate axonal and dendritic arborizations within a single neuronal population (Kang and Schuman, 1996; Lom and Cohen-Cory, 1999). However, it is unclear if BDNF driven by a specific promoter has similar effects. The exact roles of activity-driven BDNF expression in the synapse and circuit specific modulations remain elusive. BDNF’s effects are spatially confined within single neurons, or even different subcellular domains (Horch and Katz, 2002; Horch, 2004; English et al., 2012). An understanding of local signaling functions at the level of single neurons for specific BDNF promoters is essential in defining its roles in modulating neural circuitry.

We sought to contribute to this area by using layer IV circuits formed by spiny stellate cells within the mouse whisker-barrel system, the first sensory processing unit within the primary sensory cortex. In this circuit, the contribution of cell types and their different synaptic components to sensory perception/transmission can be clearly explained. Using a strain of genetically modified mice (KIV^-/-^) that exhibits relatively normal basal expression but severely reduced activity-driven BDNF expression in the cortex (Sakata et al., 2009), we have recently demonstrated a critical role of activity-driven BDNF expression in the activity-dependent modulation of GABAergic transmissions (Jiao et al., 2011). The present study examines the structural (axonal vs. dendritic) and synaptic specific (GABAergic vs. glutamatergic) properties of layer IV circuits in the KIV^-/-^ mice. In this study, we focused on spiny stellate cells within the mouse somatosensory cortex, in which previous studies have demonstrated circuit-wide changes in response to the manipulation of sensory experiences (Feldman et al., 1999; Feldman and Brecht, 2005; Holtmaat et al., 2006; Feldmeyer et al., 2013). Our results indicate that the actions of activity-driven BDNF are highly specific to the presynaptic organization of GABAergic synapses and dendrites, particularly dendritic spines, but not the formation of intracortical glutamatergic axonal arborizations and synapses. Thus, we postulate that the role of activity-driven BDNF expression is to specifically modulate the computational ability of circuits that relate to gain control; rather than the basic wiring of the circuits. These results support the idea that BDNF signaling at the level of individual neurons is highly specific, and understanding the specificity of BDNF is central to understanding how BDNF is involved in the modulation of development, maintenance, and plasticity of neural circuitry and neural basis underlying neurodevelopmental disorders involving BDNF.

## MATERIALS AND METHODS

All experiments using mice were approved by the IACUC committee of the University of Wyoming.

### BRAIN SLICE PREPARATIONS, ELECTROPHYSIOLOGICAL RECORDINGS

Mice were deeply anesthetized with isoflurane and decapitated. The brains were quickly removed and placed into cold (∼4^∘^C) oxygenated slicing medium containing (in mM): 2.5 KCl, 1.25 NaH_2_PO_4_, 10.0 MgCl_2_, 0.5 CaCl_2_, 26.0 NaHCO_3_, 11.0 glucose, and 234.0 sucrose. TC slices were prepared according to methods described by Agmon and Connors (1991). Tissue slices (300–400 μm) were cut using a vibratome (TPI, St. Louis, MO, USA), transferred to a holding chamber, and incubated (35^∘^C) for at least 1 h. Individual slices were then transferred to a recording chamber, fixed to a modified microscope stage, and allowed to equilibrate for at least 30 min before recording. Slices were minimally submerged and continuously superfused with oxygenated physiological saline at the rate of 4.0 ml/min. The physiological perfusion solution contained (in mM): 126.0 NaCl, 2.5 KCl, 1.25 NaH_2_PO_4_, 1.0 MgCl_2_, 2.0 CaCl_2_, 26.0 NaHCO_3_, and 10.0 glucose. Solutions were gassed with 95% O_2_/5% CO_2_ to a final pH of 7.4 at a temperature of 35 ± 1^∘^C. The method for identification of the barrel subfield in living TC slices was described in earlier studies (Sun et al., 2006). A low-power objective (2.5×) was used to identify barrels and thalamic nuclei, and a high-power water immersion objective (60×) with Nomarski optics and infrared video was used to visualize individual neurons. Recording pipettes were pulled from capillary glass obtained from World Precision Instruments (M1B150F-4), using a Sutter Instrument P80 puller, and had tip resistances of 2–5 MΩ when filled with the intracellular solutions below. A Multi-Clamp 700B amplifier (Axon Instruments, Foster City, CA, USA) was used for voltage-clamp and current clamp recordings. Patch pipette saline was modified according to Brecht and Sakmann (2002) and composed of (in mM): 100 K-gluconate (or Cs-gluconate for IPSC recordings), 10.0 phosphocreatine-Tris, 3.0 MgCl_2_, 0.07 CaCl_2_, 4 EGTA, 10.0 HEPES, 4.0 Na_2_-ATP, and 1.0 Na-GTP, pH adjusted to 7.4 and osmolarity adjusted to 280 mOsm. Neurobiotin (0.5%; Vector Labs) was added to the patch pipette solution. Data were accepted for analysis when access resistance in whole-cell recordings ranged from 15 to 35 MΩ, and was stable (<25% change) during the recording. The resting membrane potential and the resting input resistance of the cell was also monitored to ensure a stable baseline recording. Current and voltage-clamp protocols were generated using PCLAMP9.2 software (Axon Instruments). A sharpened bipolar tungsten electrode, placed at ∼200 μm away from recorded cells in the cortical layer IV, was used to activate intracortical fibers. eEPSCs were evoked in the presence of a cocktail ACSF solution containing GABA_A_ antagonist picrotoxin (50 μM) and low concentration of AMPA/kainate receptor antagonist 2,3-dihydro-6-nitro-7-sulfamoyl-benzo (F) quinoxaline (NBQX; 0.05 μM) to reduce excitation and prevent hyperexcitability (Kumar and Huguenard, 2003). Evoked EPSCs events were detected using Clampfit®; event detection function with either ‘threshold detection’ or ‘template detection’ method.

### CHEMICALS

AMPA antagonist GYKI 52466 hydrocloride, [1-(4-aminophenyl)-4-methyl-7, 8-methylenedioxy-5H-2, 3-benzodiazepine] (Sigma-Aldrich, St. Louis, MO 63178, USA); 2,3-dihydro-6-nitro-7-sulfamoyl-benzo (F) quinoxaline (NBQX, Tocris, Ellisville, MO 63021, USA), DL-AP5 (Tocris), Picrotoxin (Tocris).

### VARIANCE–MEAN (V–M) ANALYSIS

Variance–mean (V–M) analysis was conducted based on Oleskevich et al. (2000). Briefly, Synaptic events were evoked in different extracellular Ca^2+^/Mg^2+^ ratios (Silver et al., 1998, 2003; DiGregorio et al., 2002; Lawrence and McBain, 2003). Although there is a small differences in the concentration of the divalent cation, this is in sufficient to cause significant changes in the excitabilities (Hanck and Sheets, 1992). Next three parameters were used to describe synaptic function: the probability of vesicle release (Pr); the mean amplitude of the synaptic response to a vesicle of release (Qw); and the number of release sites (N). These parameters were obtained from the relationship between the variance and the mean of a post-synaptic amplitude recorded under various release probability conditions (Silver et al., 1998; Reid and Clements, 1999). When the V–M plot showed a typical parabolic plot y = Ax-Bx^2^ (1). The following two equations were used to calculate the average synaptic parameters: Q_w_ = A/(1+CV_I_^2^) (2); P_rw_ = X(B/A)(1+CV_I_^2^) (3); and N_min_ = 1/B (4). When the release probability was low (<0.3), the V–M relationship was approximately linear, then the plot was analyzed with the linear equation of Y = Ax (5). This would permit an estimation of Qw using equation (2), however, P_rw_ and N_min_ could not be estimated under this conditions.

### NEURONAL RECONSTRUCTION AND MORPHOMETRIC ANALYSIS

Individual spiny stellate cells were labeled via intracellular loading neurobiotin during a 30–60 min whole-cell recording session. Brain slices (350 μm) were subsequently histologically processed and mounted onto microscopy slides using methods described earlier (Young and Sun, 2009). The dendritic and axonal arbors of each spiny stellate cell were digitally traced using Neurolucida®; under 100X oil-immersion objectives. Standard morphometric analysis (e.g., Sholl analysis, polar histogram) was conducted using Neurolucida Explorer®; program, as described earlier (Young and Sun, 2009). Shrinkage related errors was not corrected.

### IMMUNOHISTOCHEMISTRY

Brains were post-fixed after perfusion in 4% paraformaldehyde at 4^∘^C overnight, cryoprotected in 30% sucrose for 2 days, frozen, and cut into 30 μm thick cryostat sections. Free-floating sections were then stained for antibody-DAB as follows: sections were rinsed in PBS, incubated for 30 min in 0.5% H_2_O_2_ in PBS, 2 min × 10 min PBS washes, incubated for 2 h at room temperature in PBS with 0.3% Triton X-100, 0.05% Tween, and 4% normal goat serum, and incubated overnight at 4^∘^C in PBS containing 0.2% Triton X-100 and primary antibodies directed against: BDNF (1:500, Santa Cruz Biotechnology, sc-546). Sections were then rinsed two times in PBS, incubated at room temperature for 90 min in PBS containing biotinylated goat anti-rabbit IgG (Vector labs), and finally incubated overnight at 4^∘^C in Vectastain ABC kit (Vector Labs). Sections were then rinsed two times in PBS, developed in 50 mM TBS containing 0.04% 3,3′-diaminobenzidine tetrahydrochloride (DAB, Sigma, St. Louis, MO, USA) and 0.012% H_2_O_2_ washed two times with TBS, mounted onto glass slides, dehydrated, cleared, and coversliped. 3-D neuron models were reconstructed from stained cells using the Neurolucida system (MicroBrightField Inc., Williston, VT, USA) and a bright-field light microscope (Carl Zeiss MicroImaging Inc., Thornwood, NY, USA). Reconstructed neurons were quantitatively analyzed with NeuroExplorer (MicroBrightField Inc.).

### MOUSE BREEDING AND GENOTYPING

The KIV^+/-^ mice were crossed again to generate F2 homozygous KIV^-/-^ and litter mate wild-type KIV^+/+^ mice. Mouse genotyping methods were described earlier by Sakata et al. (2009).

### STATISTICS

Upon group divisions, data was compared across groups using *t*-test, and/or single factor analysis of variances (one way-ANOVA) tests, followed by Tukey’s HSD test in order to determine intergroup significance. *p* < 0.05 was considered to be significantly different. In some experiments, *t*-test or Kolmogorov–Smirnov test was used as well.

## RESULTS

**FIGURE 1:**
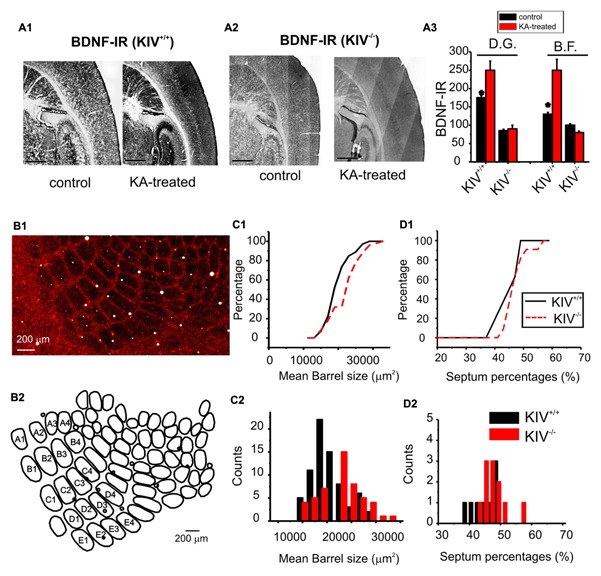
**Intact barrel structure with reduced activity-driven expression of BDNF in KIV ^**–**/**–**^ mice. (A)** Photograph of BDNF-IR in thalamocortical sections of kainic acid treated (right) vs. control (left) wild-type (A1) and mutant (KIV^-/-^, A2) mice. (A3) the intensity of BDNF-IR was measured along lines across the barrel field (BF) and hippocampus dentate gyrus area (DG), respectively. *n* = 4 mice in each group. Scale bars: 1 mm. **(B)** Photograph of cytochrome-C stained BF in a flattened tangential section across layer IV of somatosensory cortex of wild-type mice (B1). The BF was re-constructed using Neurolucida^®^, from which the morphometric data of BF was analyzed (B2). **(C)** The size of individual barrels within the primary mystacial BF (PMBF A1 though E4) was significantly larger in the mutant, *N*=4 in each group **(C)**. **(D)** In contrast, the septum percentage within PMBF did not show significant differences. The measurements were made from 6 pairs of littermate control (*n*= 6) and mutant (*n*= 6) mice, respectively.

Activity-driven BDNF-expression is abolished in the prefrontal cortex, hippocampus, and neocortex in the KIV^-/-^ mice (Sakata et al., 2009). To further confirm this in the barrel cortex, we applied kainic acid (KA) *in vivo* (30 mg/Kg). Within the first hour of application, four out five treated animals developed seizures evolving into recurrent generalized convulsions and were used for histology experiments. The mice were euthanized at 4 h post-treatment, and BDNF level was assessed using immunohistochemistry (**Figure 1A**). In wild-type mice, KA significantly increased BDNF protein levels in both the hippocampus dentate gyrus (DG) area and in the barrel field (BF). In contrast, KA did not increase BDNF levels in these two areas in the KIV^-/-^ mice (**Figure 1A3**). These results indicated that activity-driven BDNF expression in the forebrain is abolished in the KIV^-/-^ mice. We then examined the anatomical remodeling occurring within the BF of the KIV^-/-^ mice. Flattened tangential cortical slices were prepared for cytochrome-C staining to label the BF (Wong-Riley and Welt, 1980). The entire BF was reconstructed using Neurolucida®; and analyzed using Neurolucida Explorer®; (e.g., **Figure 1B**). Our results showed that there was a significant increase in the size of individual barrels within the major mystacial whisker barrels of KIV^-/-^ mice (*p* < 0.01, **Figure 1C**), but the barrel/septum ratio was unchanged (**Figure 1D**). Therefore, we conclude that the development and maintenance of major anatomical organization in the BF does not require activity-driven BDNF.

### DENDRITIC MORPHOLOGY

**FIGURE 2:**
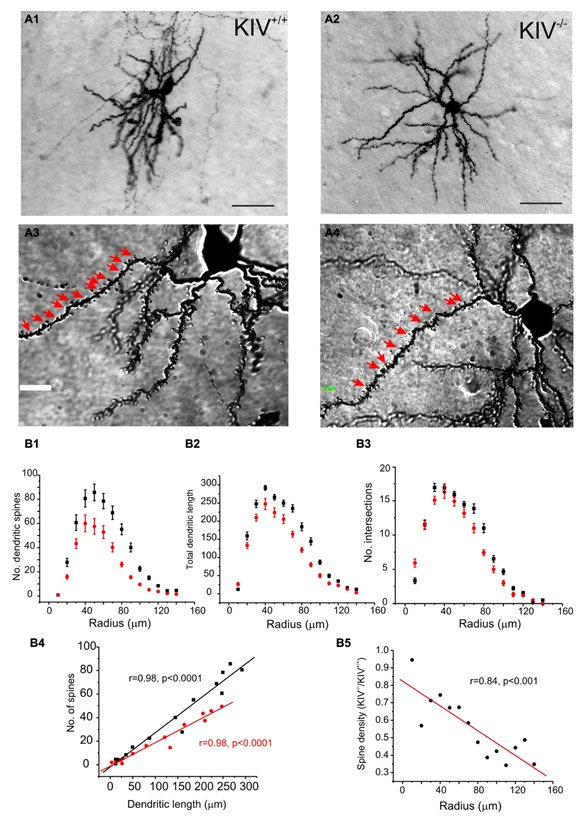
**Altered dendritic morphology in KIV^**–**/**–**^ mice. (A)** Photomicrograph of a pair of neurobiotin labeled spiny stellate cells from littermate wild-type (A1,A3) and KIV^-/-^ mice (A2,A4), respectively. Scale bar in (A1,A2) 10 μm; Scale bar in (A3,A4) 2 μm. White arrows in (A3,A4) dendritic spines. **(B)** Sholl analysis was performed to compare the distribution of dendritic spines (B1), the distribution of total dendritic length (B2), as well as the no. of intersections (B3) along different dendritic radiums from the soma. (B4) The average no. of dendritic spines was plotted against its dendritic length in wild-type mice (black squares) and KIV^-^/^-^ mice (red circles). (B5) the spine density in wild-type vs. KIV^-^/^-^ mice was plotted against the dendritic radius from the soma. *n*= 25 and 19 wild-type and mutant spiny stellate cells, respectively.

The morphological analysis was based on 44 neurobiotin labeled intact layer IV spiny stellate cells, which consisted of 25 wild-type neurons (KIV^+/+^) and 19 mutant neurons (KIV^-/-^). Cells with truncated dendrites were removed from the analysis. Spiny stellate cells were identified based on enriched dendritic spines and the absence of apical dendrites extending out of layer IV into supragranular layers (Staiger et al., 2004). We found that the dendritic morphology between the wild-type and mutant spiny stellate neurons was different in several ways. (1) The total number (**Table 1**) and density of spines on each dendrite (**Figure 2**) were significantly reduced (**Table 1**). (2) While the dendritic branching measured by the total number of intersections was unchanged, the total dendritic length was significantly reduced (**Figure 2B2**; **Table 1**). The distribution of spines along the dendrites of spiny stellate cells showed a linear correlation with dendritic location: distal dendrites had far more spines than proximal dendrites. The slope of this linear correlation was reduced in the mutant (**Figure 2B4**). As a result, the reduction in dendritic spines was positively correlated with dendritic length, i.e., distal dendritic spines are reduced the most in the mutant (**Figure 2B5**).

**Table 1:** Structural analysis of dendrites between wild-type and KIV^**–/–**^ spiny stellate cells.

	Qty	Nodes	Ends	Spines	Length (μm)	Mean length	Surface (μm^2^)	Volume (μm^3^)	Polar angle (after fan in)	Polarized cells (yes = 1, no = 0)
KIV^+/+^(*n* = 25)	4.5 ± 0.3	23.1 ± 1.4	28.0 ± 1.4	488.4 ± 64	1818.6 ± 90	438.1 ± 32	3724.0 ± 281	691.4 ± 86	126.9 ± 6.8	0.9 ± 0.1
KIV^-/-^ (*n*= 19)	4.4 ± 0.0	18.9 ± 0.1	239 ± 0.25	288.1 ± 42	1527.3 ± 96	384.0 ± 37	2828.8 ± 244	457.3 ± 56	154.2 ± 8.5	0.5 ± 0.1
*p* value	0.8	0.1	0.2	**0.04**	**0.01**	0.2	**0.01**	**0.03**	**0.04**	**0.01**

*QTY, quantity; Ends, dendritic endings; length, total dendritic length; mean length, mean dendritic length. Mean ± Standard error (SE) of the data is shown. *p* < 0.05 is considered statistically significant.*

**FIGURE 3:**
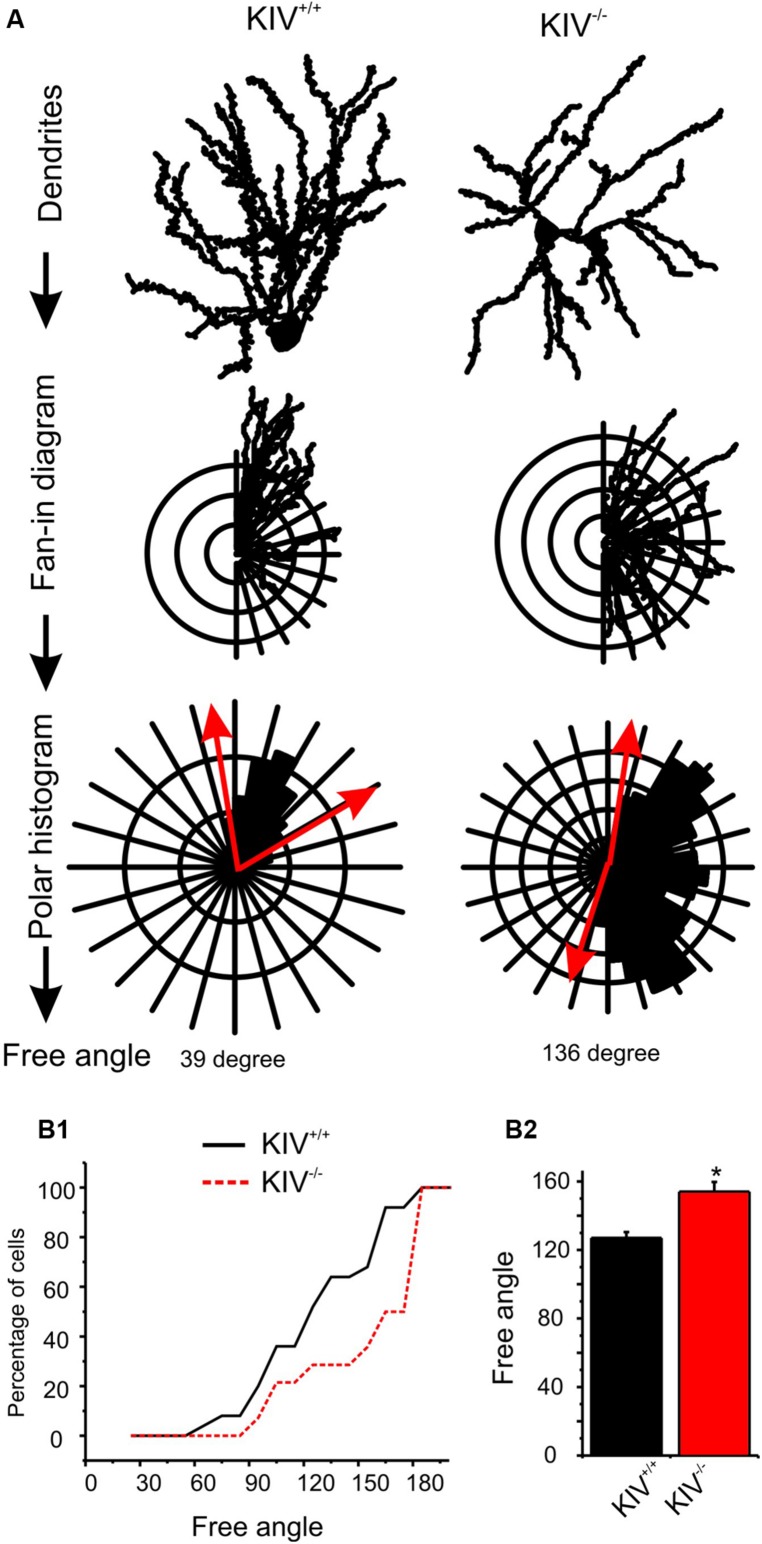
**Altered dendritic asymmetry in KIV^**-/-**^ mice. (A)** Graphs illustrated the processes where the distribution of dendritic arbors was converted into a polar histogram, from which a free angle was derived. **(B)** Free angles, an objective way to assess the asymmetric feature of dendritic arbors, were significantly larger in KIV^-/-^ mice (red, *p* < 0.05), indicating a reduced dendritic asymmetry.

The majority of wild-type spiny stellate cells showed an asymmetric dendritic branching pattern: the distribution of dendrites confined within a barrel and the soma was located near the barrel border (Staiger et al., 2004; Egger et al., 2008). This asymmetric distribution of dendritic arbors correlate with the experience-dependent refinement process (Fox and Wong, 2005). Using a method described earlier by Egger et al. (2008), we examined if dendritic asymmetry was affected in the mutants. Indeed, there were significant reductions in asymmetry in the mutant neurons, with a higher percentage of cells showing reduced asymmetry (**Figure 3**; **Table 1**). Thus, the dendritic morphology of spiny stellate cells was significantly affected by the disruption of activity-driven BDNF expression, suggesting the maintenance of normal dendritic morphology and spine density requires activity-driven BDNF. Basal level of BDNF expression is insufficient to achieve this role.

### AXONAL ARBORS

**FIGURE 4:**
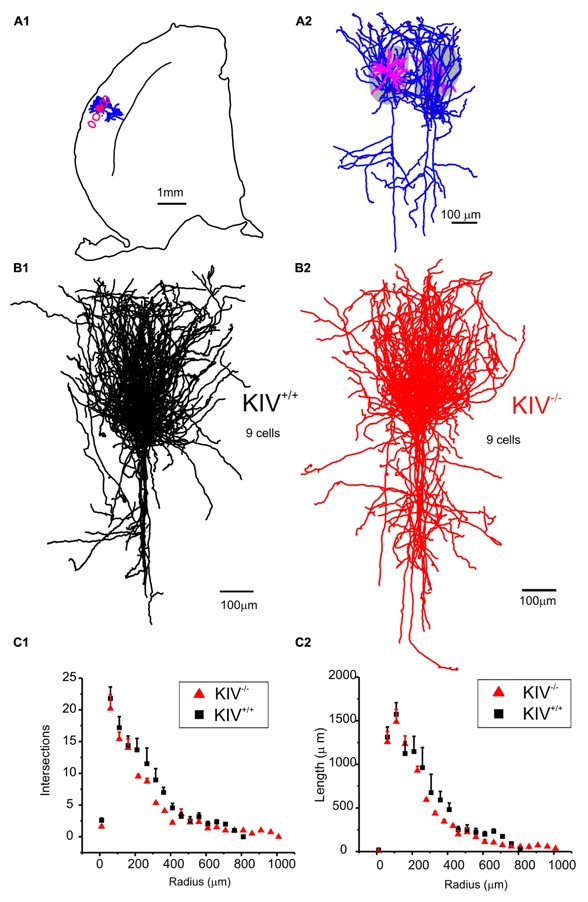
**Axonal morphology was unaltered in KIV^**-/-**^ mice. (A)** An example of a pair of reconstructed wild-type spiny stellate cells, located in neighboring barrels (grey areas in A2 and red contour in A1). Note the typical distribution of axonal arbors (blue) within layer IV barrels with projections toward layer II/III, and downward projections toward the white matter. Dendrites (magenta) are largely confined within a single barrel. **(B)** Reconstructed axonal arbors from 9 pairs of wild-type (B1, black) and littermate mutant neurons (B2, red) were superimposed at the soma location. **(C)** Sholl analysis was performed to compare the distribution of no. of intersections (C1), and the distribution of total axonal length (C2) along different radius from the soma in 21 wild-type and 10 mutant neurons, respectively. No statistical differences between WT and KIV mice.

Intracortical axonal collaterals between layer IV cells contribute profusely to the excitatory synaptic inputs in spiny stellate cells (Feldmeyer et al., 1999). The morphological analysis of axonal arbors was based on 31 neurobiotin labeled, relatively intact layer IV spiny stellate cells. These consisted of 9 out of 21 wild-type neurons (KIV^+/+^) and 9 out of 10 mutant neurons (KIV^-/-^) that were located at least 100 μm below the surface of a 350 μm brain slice. The axonal arbors of both the mutant and wild-type spiny stellate cells showed typical patterns as previously described (Staiger et al., 2004; Egger et al., 2008). Briefly, the main stem of axons originated from the basal direction of the soma and extended toward the white matter. Within layer IV, the main stem of axons gave rise to abundant recurrent collaterals that centered on the barrels and extended to layer II/III barrels (**Figure 4A**). Cells with truncated axonal arbors, i.e., severe deviation from the typical axonal morphology, were removed from analysis. The morphometric data and Sholl analysis showed that there were no significant differences in either aspects of axonal morphologies, which include both the total length and the branching patterns (**Table 2**; **Figures 4B,C**). Therefore, in contrast to the dendrites, the morphology of axonal arbors of spiny stellate cells is independent of the activity-driven BDNF expression. The basal level of BDNF expression is therefore sufficient to the development and maintenance of intact axonal arbors in these cells.

**Table 2:** Structural analysis of axonal arbors between wild-type and KIV^**–/–**^ spiny stellate cells.

	Nodes	Ends	Length (μm)	Mean length (μm)	Surface (μm^2^)	Volume (μm^3^)
KIV^+/+^ (*n*= 9)	52.3 ± 8.7	54.0 ± 8.9	6174.4 ± 1013	6119.7 ± 1045	6270.3 ± 1314	608.8 ± 143
KIV^-^**^/^**^-^(*n*= 9)	58.1 ± 6.8	60.2 ± 6.8	6944.0 ± 834	6435.7 ± 627	6516.4 ± 946	554.2 ± 149
*p* value	1.0	0.9	0.5	0.8	0.8	0.7

### GABAergic TRANSMISSION

**FIGURE 5:**
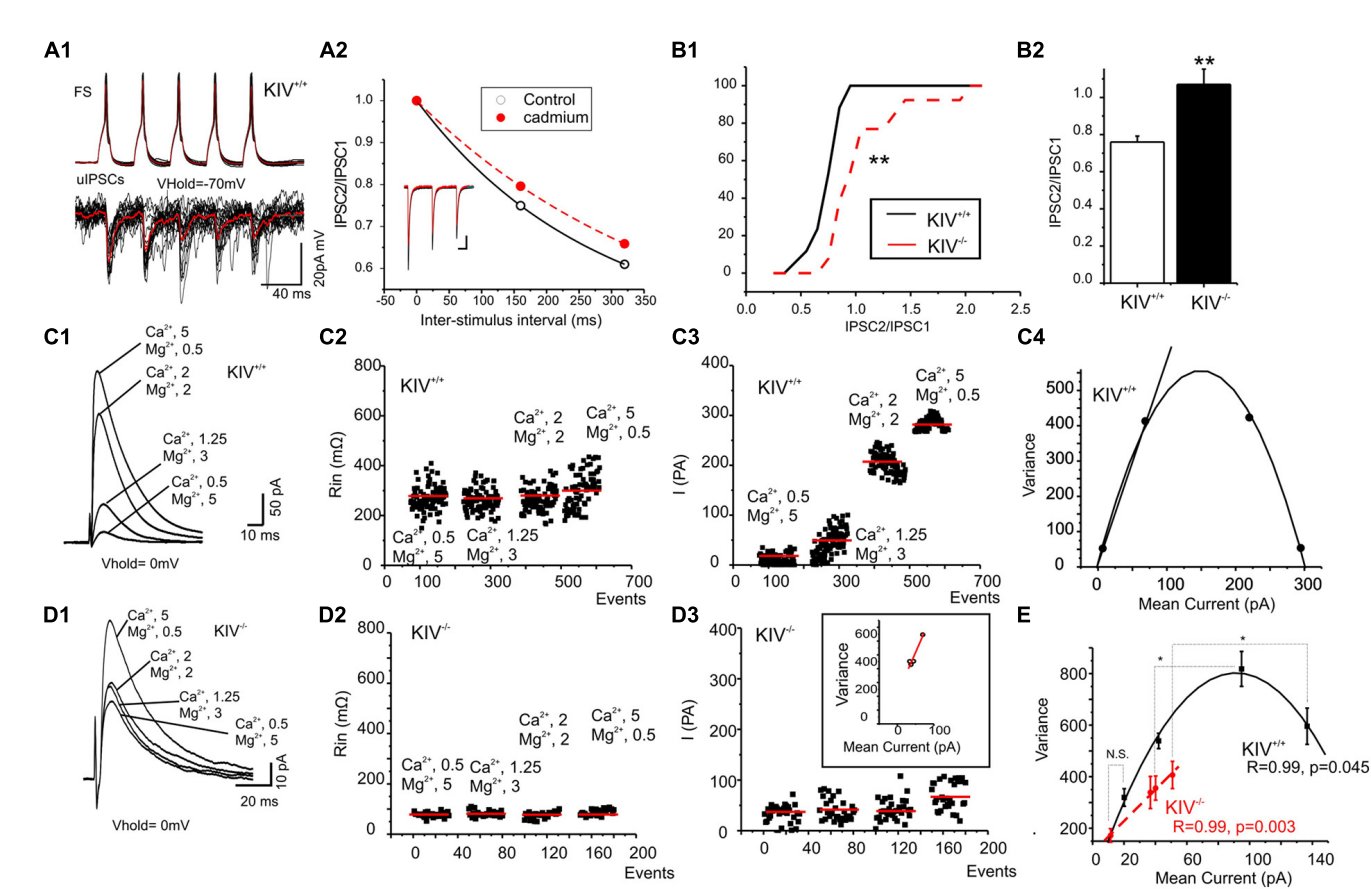
**Altered presynaptic GABAergic transmission in KIV^**–/–**^ mice. (A)** Paired recordings from a pair of synaptic connected cells in layer IV of a wild-type mouse. Repetitive action potentials (top) in a FS cell induced GABAergic uIPSCs (bottom) in the post-synaptic spiny stellate cell (A1). The averaged uIPSCs (red trace in the bottom) showed a short-term depression, which was reduced by calcium channel blocker cadmium (100 μM, A2). **(B)** The characteristic paired-pulse depression of eIPSCs in spiny stellate cells of wild-type mice was abolished in KIV^-/-^ mice. *n* = 8 cells in each group. **(C,D)** IPSCs were evoked in the presence of different extracellular Ca^2+^/Mg^2+^ ratios (C1,D1) in wild-type (**C**, *n* = 8) and KIV^-/-^ mice (**D**, *n* = 8). The input resistance (C2,D2), and amplitude (C3,D3) of each evoked IPSC event was plotted against experimental time. (C4,D4) V–M analysis of the same experiments of C1,D1. **(E)** The V–M analysis of all wild-type mice (black squares) and KIV^-/-^ mice (red circles), statistical analysis were performed between amplitude of eIPSCs evoked in the same conditions. *p < 0.05; **p < 0.01.

**FIGURE 6:**
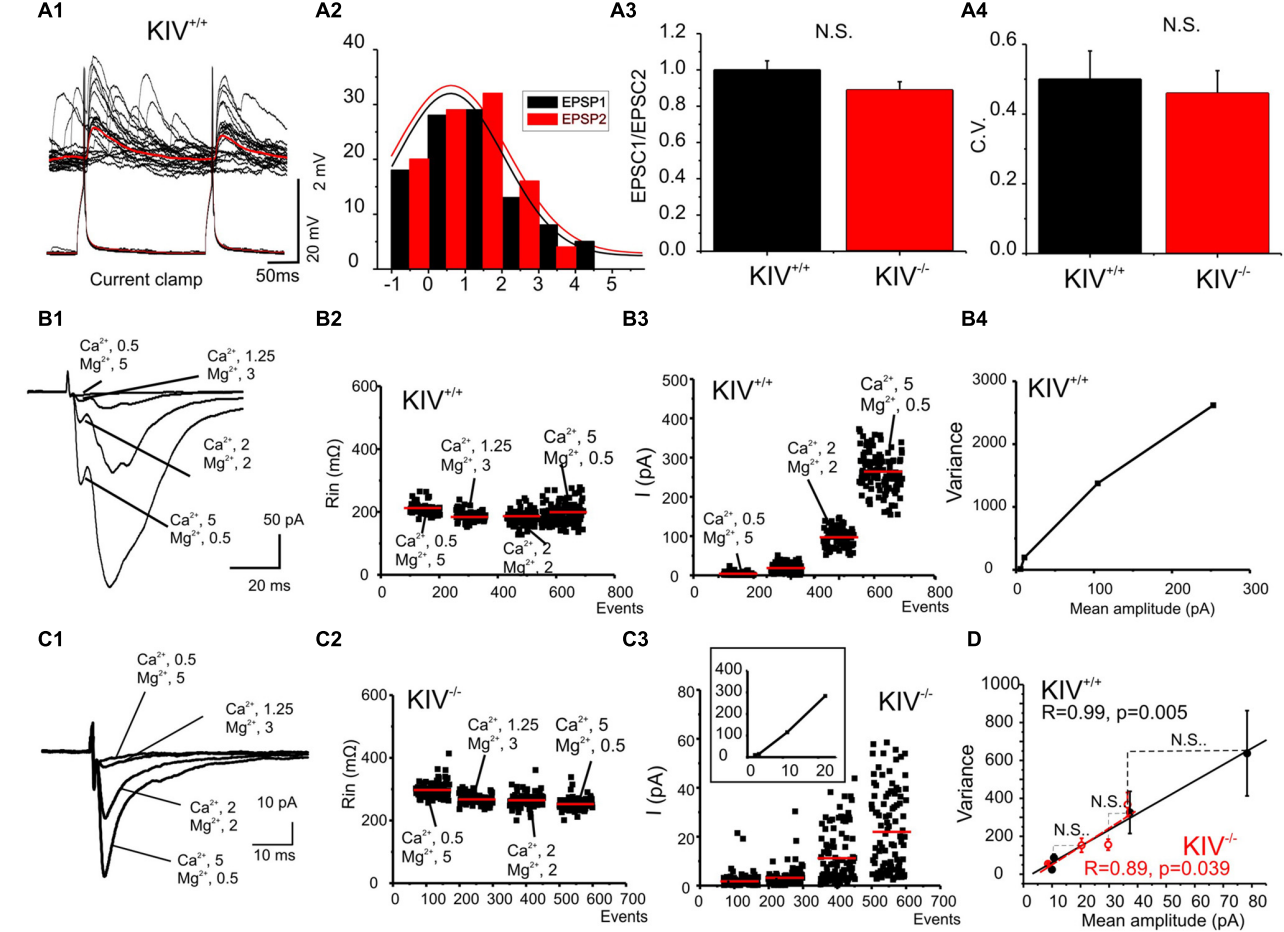
**Unaltered presynaptic glutamatergic transmission in KIV^**-/-**^ mice. (A)** Paired recordings from a pair of synaptic connected cells in layer IV in a wild-type mouse. Repetitive action potentials (bottom) in a spiny stellate cell induced uEPSPs (top) in a post-synaptic spiny stellate cell (A1). The averaged uEPSPs (top red trace) showed little short-term depression (A2). (A3,A4) There were no significant differences between the paired-pulse ratios (A3) and CV (A4) of eIPSCs in spiny stellate cells of wild-type vs. mutant (KIV^-/-^) mice, *n* = 9 cells in each group. **(B,C)** EPSCs were evoked in the presence of different extracellular Ca^2+^/Mg^2+^ ratios (B1,C1) in wild-type **(B)** and KIV^-/-^ mice **(C)**. The input resistance (B2,C2), and amplitude of each eIPSC event was plotted against experimental time. (B4) V–M analysis of the same experiments of B1. **(D)** The V–M analysis of all wild-type mice (black squares, *n* = 9) and KIV^-^/^-^ mice (red open circles, *N* = 9). The linear fitting curves were essentially overlap between wild-type and KIV^-^/^-^ mice. Statistical analysis were performed between amplitude of eIPSCs evoked in the same conditions. There were no significant differences between the wild-type and KIV^-/-^ mice.

We previously reported the disturbance of excitation–inhibition (E–I) balance in the KIV^-/-^ mice (Jiao et al., 2011). To further understand how specific synapses are affected and the underlying synaptic mechanisms, we compared GABAergic transmissions within spiny stellate cells from wild-type and KIV^-/-^ mice. Paired recordings were initially made from wild-type mice. The unitary IPSCs (uIPSCs) onto spiny stellate cells showed a strong paired-pulse depression that was reduced by calcium channel blocker cadmium (100 μM, **Figure 5A2**), suggesting that inhibitory synapses were high release probability synapses (Xu-Friedman and Regehr, 2000; Silver et al., 2003). Next, evoked IPSCs (eIPSCs) were induced by an extracellular tungsten stimulating electrode located near the recorded cells (<100 μm), in the presence of a cocktail solution containing NBQX (10 μM) and AP-5 (100 μM) to block AMPARs and NMDARs. Consistent with the results of paired recordings, the eIPSCs showed a typical paired-pulse depression in the wild-type mice, whereas the paired-pulse depression was abolished in the mutant mice (**Figure 6B2**). This data suggests that the calcium-dependent release of GABA was compromised in the mutants. We next studied the properties of eIPSCs under different calcium and magnesium ratios in the extracellular solution (Silver et al., 2003). VM analysis was performed based on the methods described previously (Silver, 2003). As shown in **Figures 5C2,D2**, the change of extracellular calcium and magnesium ratios did not induce significant changes in the input resistance (Rin) of the recorded cells. The V–M plot of eIPSCs in the wild-type mice showed a feature that was consistent with the high release probability (Pr) synapses: when the Pr was low (e.g., at 0.5/5 mM Ca^2+^/Mg^2+^), the trial to trial variance of eIPSC amplitude was low; when the Pr was moderate (at 1.25/3 or 2/2 mM Ca^2+^/Mg^2+^), the trial to trial variability was high; when the Pr was high (at 5/0.5 mM Ca^2+^/Mg^2+^), almost all sites released GABA after every stimulus and the eIPSC amplitude variance was low (**Figure 5C**). Therefore, the V–M plot of GABA release in the wild-type neurons showed a typical parabolic plot y = Ax-Bx^2^ (1). The following three equations were used to calculate the average synaptic parameters: quantal content (Qw) = A/(1+CV_1_^2^) (CV, coefficient of variance) (2); release probability of the eIPSCs P_rw_ = X(B/A)(1+CV_1_^2^) (3); and number of minimum release site (N/_min_) = 1/B (4). The eIPSCs of wild-type mice were estimated to have a Qw of 14.3 ± 2.5 pA, with N/_min_ of 10 ± 2. The P_rw_ varies from 0.1 ± 0.2 at 0.5/5 mM Ca^2+^/Mg^2+^ to 1.0 ± 0.2 at 5/0.5 mM Ca^2+^/Mg^2+^. Next, we studied the eIPSCs in KIV^-/-^ mice under similar conditions and performed V–M analysis. As shown in **Figure 5D**, eIPSCs in mutant neurons did not show the typical V–M relationship that can be plotted using a typical parabolic plot. In contrast, the V–M relationship in mutants was linear, which indicated that the Pr of these synapses was always low (<0.3), with a significantly smaller Qw of 3.8 ± 1.5 pA (*p* < 0.05 vs. wild-type mice). The amplitudes of eIPSCs in the four different Ca^2+^/Mg^2+^ concentrations also showed statistical significant differences at higher Ca^2+^ concentrations (*p* = 0.3, 0.5, 0.04, and 0.01, respectively) between wild-type (17 ± 2, 45 ± 7, 91 ± 11, and 131 ± 13 pA, respectively) and KIV group (11 ± 3, 36 ± 4, 40 ± 3, and 51 ± 5 pA, respectively). The results of the V–M analysis indicate that inhibitory synaptic transmission is compromised in KIV^-/-^ mice, with significantly lower quantal content and release probabilities. These findings suggest that activity-driven BDNF expression fine-tunes the strength of intracortical GABAergic transmissions by remodeling of presynaptic calcium-dependent vesicle release features of GABAergic synapses. Thus, activity-driven expression of BDNF is required for maintaining the presynaptic features of GABAergic synapses, and the basal level of BDNF is insufficient to maintain these features.

### GLUTAMATERGIC TRANSMISSION

Intracortical unitary EPSCs between wild-type spiny stellate cells did not show the type of paired-pulse depression as seen in uIPSCs (**Figure 6A**). Next, eEPSCs were induced by placing an extracellular tungsten stimulating electrode near the recorded cells (<100 μm), in the presence of a cocktail solution containing picrotoxin (100 μM) and a low concentration of NBQX (0.1 μM) to partially block AMPARs and prevent epileptiform activity in the tissue (Kumar and Huguenard, 2001). Similar to uEPSCs, the eEPSCs did not show strong paired-pulse depression in either mutant or wild-type mice (**Figure 6A3**). The CV of the amplitudes of eEPSCs also remained similar between wild-type and KIV^-/-^ mice (**Figure 6A4**). To further examine the properties of intracortical glutamate transmission, we studied the properties of eEPSCs under different calcium and magnesium ratios in the extracellular solution (Silver et al., 2003). As shown in **Figures 6B2,C2** change of extracellular calcium and magnesium ratios did not induce significant changes in the input resistance (Rin) of the recorded cells. eEPSC VM plot showed a linear regression with similar slopes in the wild-type and mutant neurons (**Figure 6**). This indicates that the release probability (Pr) for these synapses was restricted to the lower range (<0.3), therefore a linear fit was appropriate: Y = Ax (5). We next estimated the Qw using equation (2): Q_w_ = A(1+CV^2^). Q_w_ of the KIV^-/-^ mice (7.2 ± 1.0) was similar to that of wild-type mice (7.4 ± 0.8, *p* > 0.3). The amplitudes of evoked EPSCs in the four different Ca^2+^/Mg^2+^ concentrations showed no statistical significant differences (*p* = 0.8, 0.4, 0.6, and 0.3, respectively) between wild-type (10 ± 2, 11 ± 1.5, 37 ± 7, and 78 ± 20 pA, respectively) and KIV group (9 ± 2, 20 ± 3, 29 ± 4, and 36 ± 5 pA, respectively). Our results indicated that the properties of glutamatergic EPSCs induced by local electrical stimulation were similar between the wild-type and KIV^-^/^-^ mice. These findings support the idea that excitatory transmission in the mutant is similar to the wild-type mice. Therefore, activity-driven BDNF is not required for the development/maintenance of the properties of glutamate synapses, and a basal level of BDNF appears to be sufficient to achieve this role.

## DISCUSSION

The output of neural circuits are fine-tuned according to different brain states (Hasenstaub et al., 2007), which underlies cognitive function of the brain. Abnormal balance of excitation and inhibitions underlies neurodevelopmental disorders and epilepsy (Yizhar et al., 2011; Paz et al., 2013). Understanding the relationship in how activity and BDNF interacts to generate circuit specific modulation and control cortical plasticity is imperative. Using the KIV^-/-^-GAD67-GFP^+/-^ line, allowing the visualization of GABAergic interneurons, we have previously addressed the effects of the KIV^-/-^ line on whisker-trimming induced plasticity of GABAergic synapses (Jiao et al., 2011). However, further investigations of the consequences of disrupting of activity-driven BDNF expression on the maturation of the entire neuronal network *in vivo* are necessary. This type of work has never been done for the KIV^-/-^ mutants. To our knowledge, this type of work has not been done for other mouse models of reduced BDNF (e.g., heterozygotes).

Our first major finding is that activity-driven BDNF expression in the barrel cortex is important for the maturation and/or maintenance of dendritic arborizations, especially spine densities. Although it is well established that BDNF contributes to the sculpting of dendrites and synapses (Horch et al., 1999; Horch and Katz, 2002; Gorski et al., 2003; Jin et al., 2003; English et al., 2012), the range and source of BDNF signaling underlying these functions is unclear. Data from our studies indicates that sensory activity-dependent synthesis of BDNF accounts for approximately 2/3 of the total BDNF within the barrel cortex (Jiao et al., 2011). Sensory experience drives the stabilization of new spines in the subclasses of cortical neurons and promotes the formation of new synapses (Holtmaat et al., 2008). In the BDNF heterozygote mouse, BDNF expression is reduced to less than half of wild-type mouse. However, both the spine density, spine morphology, and synaptic vesicle distribution is indistinguishable from wild-type controls (Genoud et al., 2004). In contrast, in the *bdnf-*KIV mice, the reduction in BDNF protein is similar to the BDNF heterozygote mouse, yet, both spines are severely reduced (**Figure 2**). More importantly, the dendritic asymmetry, a feature highly unique to the spiny stellate cells (Staiger et al., 2004; Egger et al., 2008), were shown to be regulated via experience-dependent process (Fox and Wong, 2005), as was indeed reduced in the KIV^-/-^ mice. Thus, our data described here indicates, for the first time, that constitutive BDNF is insufficient to maintain the spine density and normal dendritic arborizations in the sensory cortex, and activity-driven BDNF is responsible for the stabilization of dendritic spines.

In contrast to the dendrites, activity-driven BDNF expression is *NOT* required for the maturation and/or maintenance of intracortical glutamatergic axonal arbors *in vivo*. Previous studies conclude that BDNF’s modulation occurs both within axonal and dendritic compartments (Cohen-Cory and Fraser, 1995; Cabelli et al., 1997), and is mediated by protein synthesis in both compartments as well (Kang and Schuman, 1996). BDNF is essential for the outgrowth and activity-dependent remodeling of axonal arbors *in vivo* (Hu et al., 2005; Jeanneteau et al., 2010). However, it is unclear whether this requires constitutive and/or activity-driven BDNF. The axonal arbors of layer IV spiny stellate cells shows stereotyped organization in a manner that facilitates thalamocortical relay onto layer II/III cells (Staiger et al., 2004; Egger et al., 2008). Studies have shown that the intracortical axonal branches in layer 2/3 are modulated by sensory manipulations (Bruno et al., 2009). Our data suggests that there are no significant differences in axonal arbors of layer IV cells between wild-type mice and mice lacking activity-driven BDNF, even though the dendritic arbors showed significant differences in the same neuron. Thus, we propose that the activity-dependent remodeling of axonal arbors is *NOT* mediated by activity-driven BDNF expression. It appears that the basal level of BDNF is adequate in the development and/or maintenance of axonal arbors of adult spiny stellate cells.

Our third and perhaps most surprising conclusion is related to the subcellular specificity of the effects of activity-driven BDNF expression within layer IV. (1) presynaptic GABAergic transmissions, but not glutamatergic transmissions, are affected in KIV^-/-^ mice (**Figures 5** and **6**). (2) Within GABAergic synapses, the effects of mutation selectively affect the synaptic transmission mediated by presynaptic calcium-dependent properties (release probability, no. of release sites, and quanta content). Moreover, our previous results showed that the disruption of activity-driven BDNF expression prevented sensory deprivation-induced barrel-specific attenuations of GABAergic transmissions. In the KIV^-/-^ mice, whisker-trimming induced plasticity in inhibitory synaptic transmissions was entirely abolished (Jiao et al., 2011). In addition, there are no significant differences in the properties of miniature IPSCs between the wild-type and KIV^-/-^ mice (Jiao et al., 2011). The strong effects on dendritic spines and GABAergic transmissions vs. lack of effects on axonal arbors and glutamate transmissions appear to be paradoxical: the spine is usually associated with the establishment of glutamatergic synapses. However, it has been reported that GABAergic synapses formulate at the dendritic spines of spiny stellate cells (Knott et al., 2002). We further hypothesize that dendritic spines are subdivided into GABAergic containing and putative glutamatergic spines. Our results indicate that these GABAergic containing dendritic spines require expression of activity-driven BDNF. Our data should not be interpreted as lack of any changes associated with glutamatergic synapses. In fact, both our current data (**Figures 6B1** vs. **6C1**) and earlier data (Jiao et al., 2011), demonstrated a reduction of glutamatergic synaptic conductances in the KIV mice. It is remarkable that a significant removal of BDNF (about 2/3 of reduction in total BDNF is mediated via activity-dependent process), only produced negligible effects on presynaptic properties of intracortical glutamatergic transmissions, but severely changed the presynaptic but not post-synaptic GABAergic transmissions (this study), and completely abolished whisker-trimming induced plasticity of the GABAergic network *in vivo* (Jiao et al., 2011). The network consequences of such an effect are intriguing. While the function of intracortical glutamatergic transmissions between layer IV spiny cells is to relay sensory information onto layer II/III (Shepherd and Svoboda, 2005; Bruno and Sakmann, 2006), the function of intracortical GABAergic transmissions is to generate feed-forward inhibition (Bruno and Simons, 2002; Swadlow, 2003; Sun et al., 2006), which provides gain control over receptive field properties. Activity-driven BDNF selectively targets the feed-forward inhibition, but spares the glutamatergic transmission and wiring, which is fundamentally important for the sensory transmission.

Although BDNF has been postulated to play critical roles in neuronal circuit development and plasticity (Greenberg et al., 2009; Deinhardt and Chao, 2013), here we demonstrated a high degree of selectivity of activity regulated BDNF expression mediated effects on individual neurons within a functional network *in vivo*. The contrasting effects of genetic removal of activity-driven BDNF on dendrites vs. axonal arbors, and GABAergic vs. glutamatergic synapses, demonstrate a highly specific effect of neurotrophin in the activity-dependent sculpting of neural circuits. We postulate that the role of activity-dependent BDNF is to modulate the computational ability of circuits that relate to the gain control (i.e., feed-forward inhibition); whereas the basic wiring of circuits relevant to the sensory pathway is spared. Gain control modulation within cortical circuits has broad impact on cognitive processing attention related behavior (Kerlin et al., 2010). Cognitive behavior and mode is determined by brain states. Thus the studying of circuit alteration by endogenous BDNF provides insights into the cellular and molecular mechanisms of diseases mediated by BDNF. These results raise a number of interesting questions regarding the need to uncover the precise subcellular and molecular mechanisms of such an action. These results also shed light on the potential neural mechanisms underlying cognitive impairments within the sensory system.

## Conflict of Interest Statement

The authors declare that the research was conducted in the absence of any commercial or financial relationships that could be construed as a potential conflict of interest.
